# Poststroke fever etiology: A case report and review of the literature on probable piperacillin–tazobactam-induced drug fever and agranulocytosis

**DOI:** 10.1097/MD.0000000000050398

**Published:** 2026-08-28

**Authors:** Lijiang Wang, Wei Jin, Yuhua Wu, Menglu Zhu

**Affiliations:** a Department of Pharmacy, The Fourth Affiliated Hospital of School of Medicine, International School of Medicine, International Institutes of Medicine, Zhejiang University, Yiwu, Zhejiang, China.

**Keywords:** agranulocytosis, drug fever, leukopenia, piperacillin–tazobactam, stroke

## Abstract

**Rationale:**

Piperacillin–tazobactam is a widely used antibiotic, but its adverse effects, particularly drug fever and hematological toxicities, are often underestimated. The concurrent occurrence of drug fever and agranulocytosis may complicate the differential diagnosis of persistent or recurrent fever, particularly in patients with stroke-associated pneumonia. We report a case of probable piperacillin–tazobactam–associated drug fever and agranulocytosis and review the relevant literature.

**Patient concerns:**

A 45-year-old woman with cerebral infarction complicated by stroke-associated pneumonia received prolonged piperacillin–tazobactam therapy. During continued treatment, she developed recurrent high fever accompanied by a progressive decline in neutrophil count, with an absolute neutrophil count nadir of 0.3 × 10^9^/L.

**Diagnoses:**

After systematic evaluation of infectious and noninfectious causes, the patient was considered to have probable piperacillin–tazobactam–associated drug fever and agranulocytosis. A Naranjo Adverse Drug Reaction Probability Scale score of 7 supported a probable association between piperacillin–tazobactam exposure and the observed adverse events.

**Interventions:**

Piperacillin–tazobactam was discontinued. Granulocyte-supportive treatment, including granulocyte colony-stimulating factor, was administered, and the antimicrobial regimen was adjusted to address the possibility of concurrent or secondary infection.

**Outcomes:**

Following discontinuation of piperacillin–tazobactam, granulocyte-supportive treatment, and antimicrobial adjustment, the patient’s fever resolved and the neutrophil count gradually recovered. She was discharged in stable clinical condition, and no recurrence of fever was reported during follow-up for up to 1 year.

**Lessons:**

In patients receiving prolonged piperacillin–tazobactam therapy, recurrent or persistent fever accompanied by a declining neutrophil count should raise suspicion for drug fever and hematological toxicity. Serial blood-cell monitoring and careful evaluation of the temporal relationship between drug exposure and clinical manifestations are important for early recognition. However, infectious and other noninfectious etiologies should be systematically evaluated before attributing these findings to piperacillin–tazobactam, and causality should be interpreted cautiously when multiple therapeutic interventions are performed concurrently.

## 1. Introduction

Piperacillin–tazobactam is a widely used broad-spectrum antibiotic combination. Although hematological adverse reactions are uncommon, they encompass a broad clinical spectrum ranging from leukopenia and neutropenia to potentially life-threatening agranulocytosis. A systematic review of published case reports reported that hemolytic anemia, thrombocytopenia, and neutropenia accounted for 40.3%, 37.1%, and 19.4%, respectively, of the reported hematological adverse reactions associated with piperacillin–tazobactam.^[1]^ More recently, a retrospective study involving 8055 patients treated with piperacillin–tazobactam reported leukopenia and neutropenia in 0.51% and 0.36% of patients, respectively. Among patients with leukopenia, the mean time to onset was 17.7 ± 6.6 days; 70.7% developed neutropenia, and 7.3% experienced drug fever.^[2]^ These findings suggest that hematological toxicity is uncommon but should be considered during prolonged piperacillin–tazobactam therapy.

Although piperacillin–tazobactam-associated neutropenia and drug fever have been reported individually, their concurrent occurrence in patients with poststroke fever remains rarely described. Because persistent fever after stroke is commonly attributed to infectious or neurological causes, medication-related fever may be overlooked during clinical evaluation. This case highlights the importance of including piperacillin–tazobactam-induced drug fever and agranulocytosis in the differential diagnosis of poststroke fever, demonstrates the value of systematic causality assessment using the Naranjo scale, and emphasizes the need for routine hematological monitoring during prolonged therapy.

## 2. Case data

Female, 45 years old, was admitted to the Neurology Department on December 20, 2021, due to “reduced speech for over 1 day.” Medical history included hypertension for over 7 years, with irregular antihypertensive medication use. One day prior to admission (December 19, 2021), the patient experienced reduced speech without obvious cause upon waking, accompanied by slightly delayed responsiveness, later progressing to complete aphasia. An emergency visit to our hospital on December 20, 2021; head magnetic resonance imaging indicated multiple recent cerebral infarctions in both frontal lobes; chest computed tomography (CT) showed no significant acute signs. The Kubota Water Swallowing Test was graded 4, with an National Institutes of Health Stroke Scale score of 7 and an modified rankin scale score of 4. The clinical diagnoses were cerebral infarction and hypertension. Initial management included antiplatelet therapy with aspirin, lipid-lowering and plaque-stabilizing therapy with atorvastatin, and collateral circulation improvement with butylphthalide.

### 2.1. Hospital course

On the second day of admission (December 21, 2021), the patient developed a fever of 38.6°C during the night. Laboratory tests revealed a white blood cell count of 12.2 × 10^9^/L, with a neutrophil percentage of 77.2% and an absolute neutrophil count of 9.4 × 10^9^/L. C-reactive protein (CRP) was 22.1 mg/L, and procalcitonin was 0.025 ng/mL. Electrolytes, tumor markers, and antinuclear antibody series were all within normal limits. Considering the possibility of aspiration pneumonia due to impaired consciousness and vomiting before admission, anti-infective therapy with piperacillin–tazobactam 4.5g every 8 hours was initiated on December 22, 2021.

### 2.2. Fever and treatment response

From December 22 to 23, the patient’s maximum temperatures were 38.5°C and 38.2°C, respectively. Blood culture results on December 23 showed Gram-positive cocci; treatment with piperacillin–tazobactam was continued. The patient experienced recurrent nausea and vomiting, with imaging indicating cerebral infarction progression. Head CT (December 24) showed cerebral infarction in both frontal lobes and the basal ganglia. Magnetic resonance angiography (December 25) demonstrated extensive acute cerebral infarction in both frontal lobes, semioval center, and beside the anterior horns of the lateral ventricles. A chest CT (December 30) revealed mild exudation in the lower lobes of both lungs, pulmonary hyperplastic foci, and bilateral small pleural effusions. A follow-up chest CT on January 7, 2022, showed similar findings.

From December 24, 2021, to January 10, 2022, the patient was lethargic with recurrent fever, cough, and night sweats. Treatment with piperacillin–tazobactam was continued throughout this period.

### 2.3. Critical event and management

**Figure 1.**
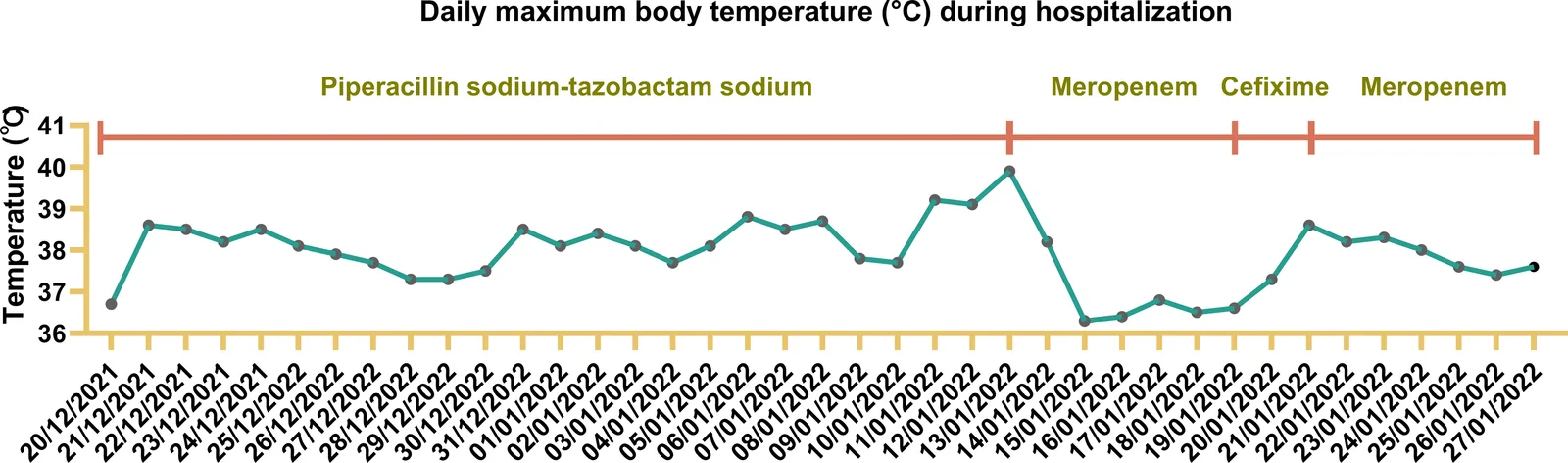
Temperature trends during hospitalization.

**Figure 2.**
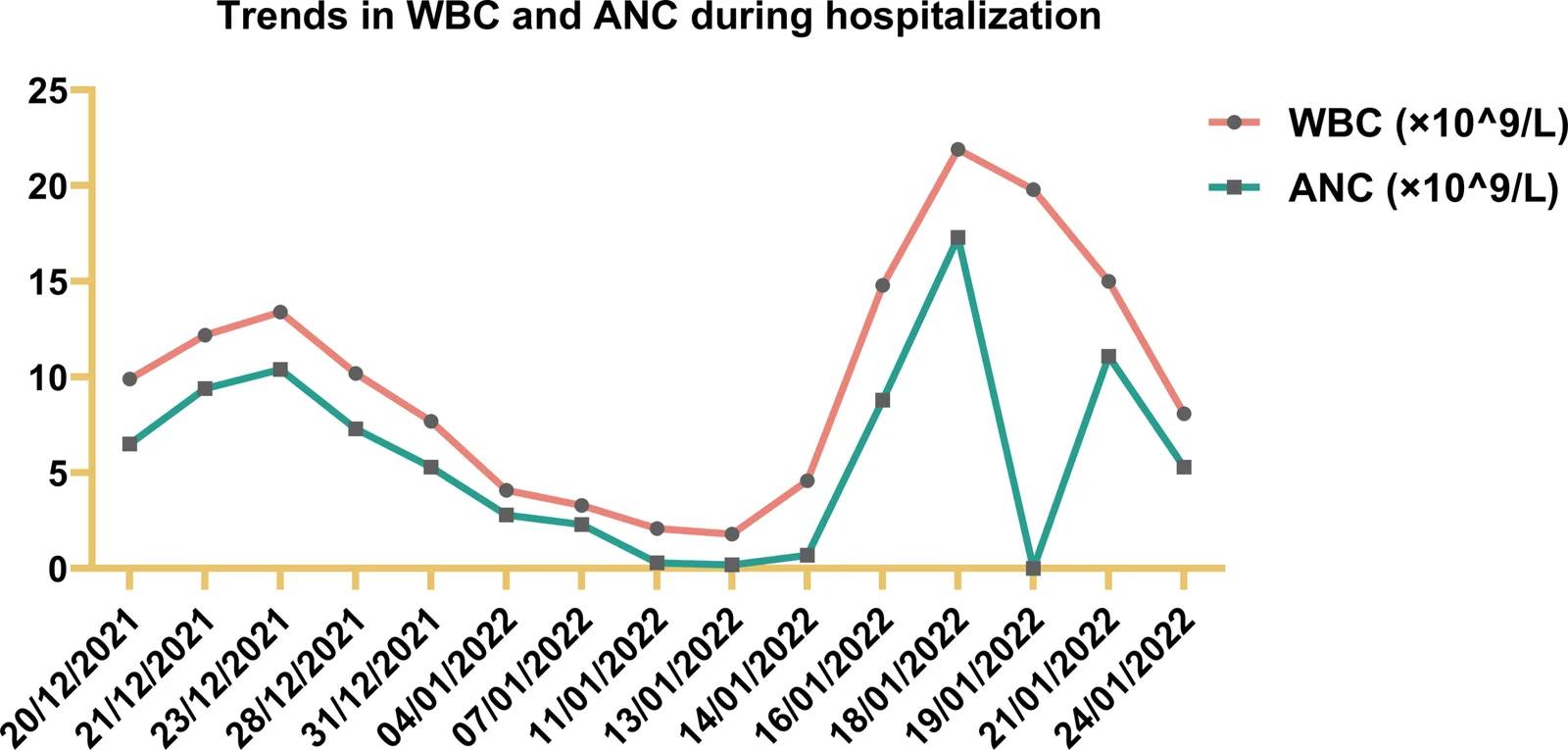
Trends in WBC and ANC during hospitalization. ANC = absolute neutrophil count, WBC = white blood cell.

**Figure 3.**
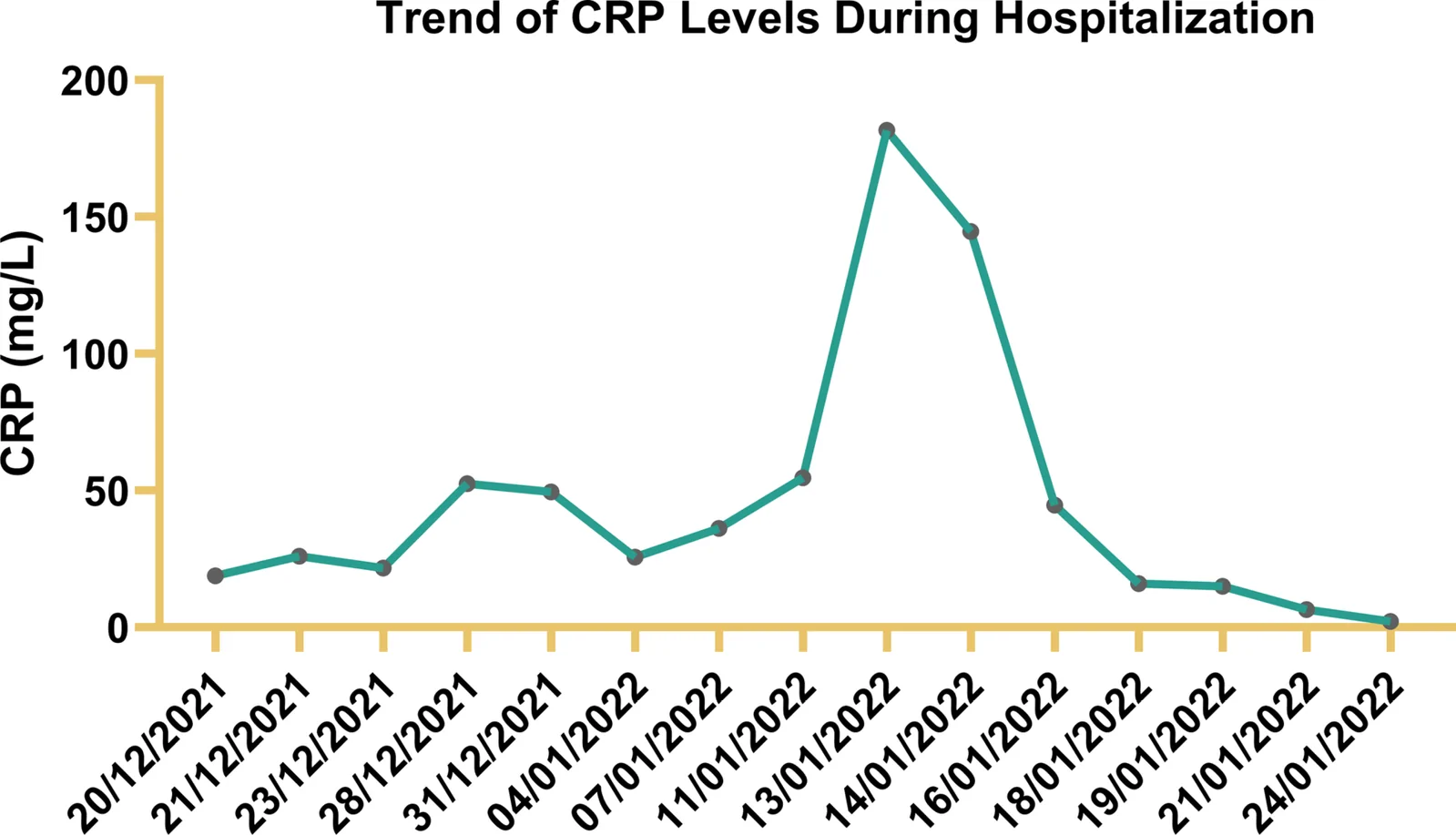
Trend of CRP levels during hospitalization. CRP = C-reactive protein.

**Figure 4.**
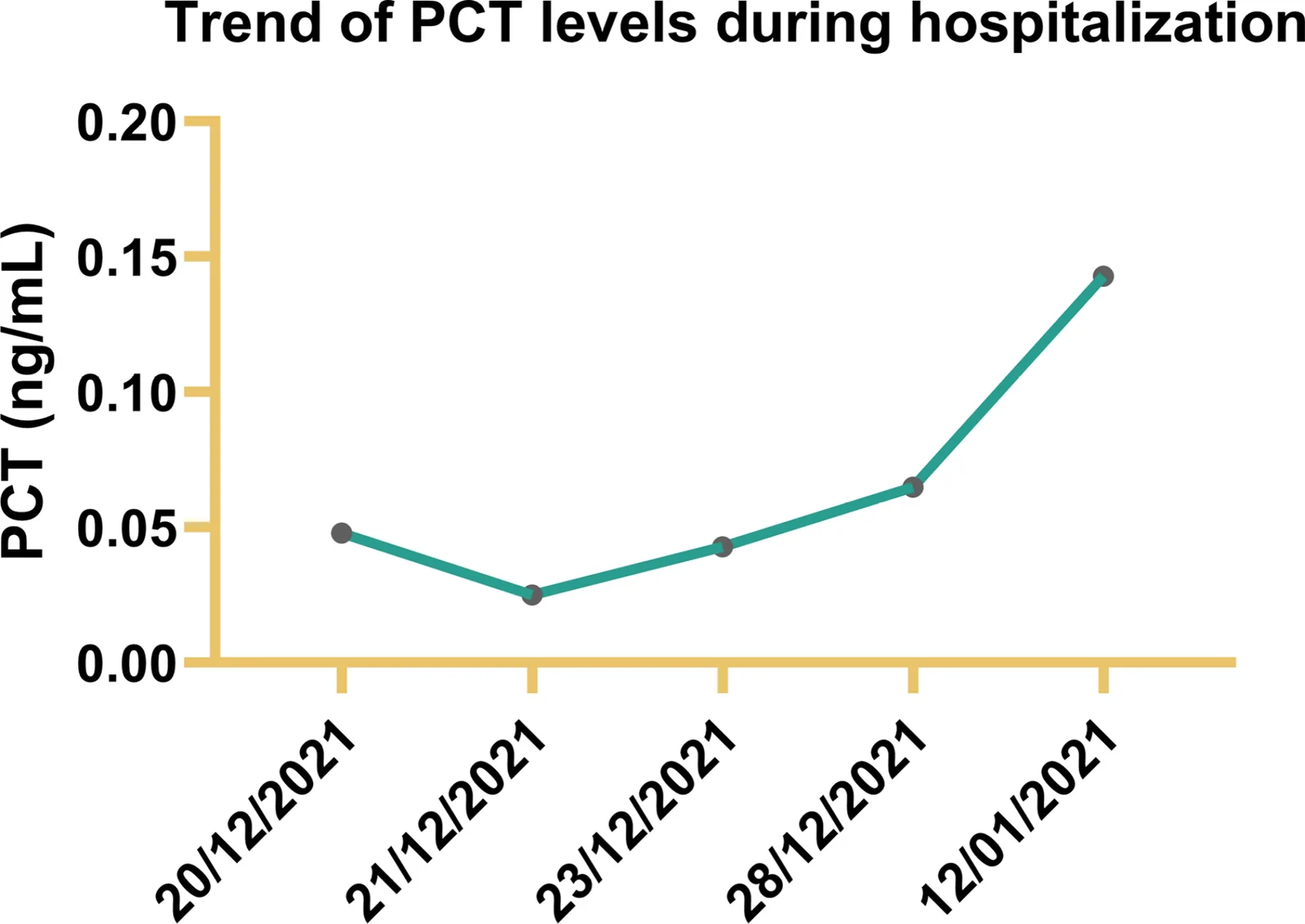
Trend of PCT levels during hospitalization. PCT = procalcitonin.

**Figure 5.**
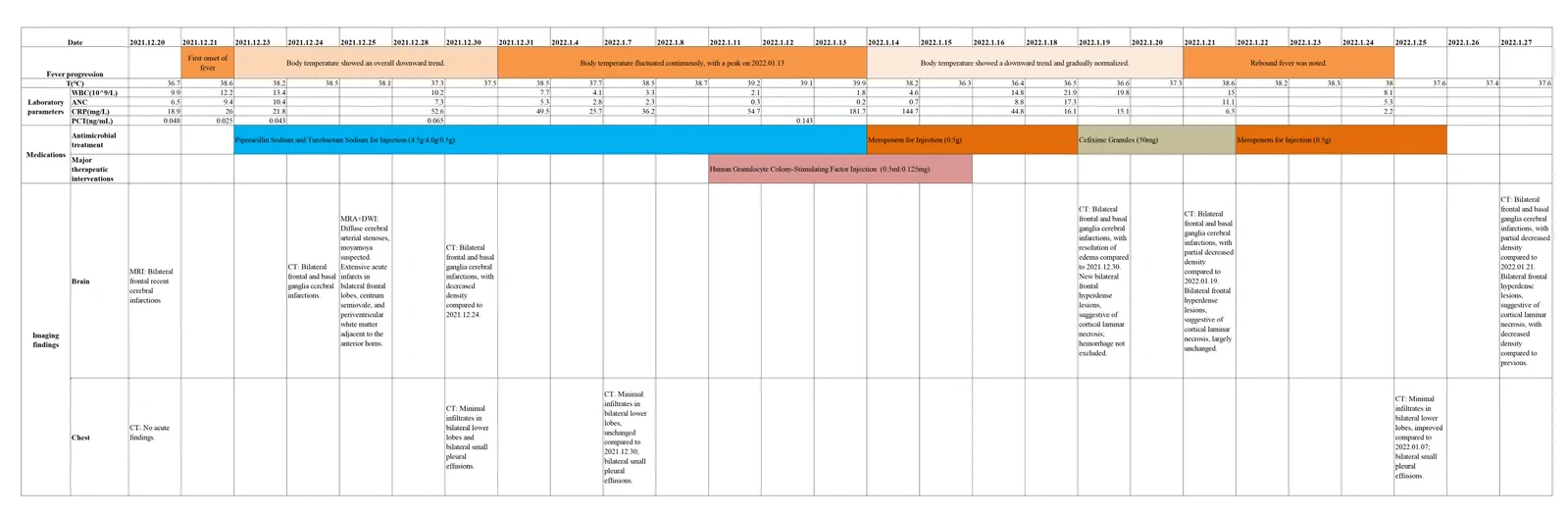
Comprehensive clinical timeline. ANC = absolute neutrophil count, CRP = C-reactive protein, CT = computed tomography, DWI = diffusion-weighted imaging, MRA = magnetic resonance angiography, MRI = magnetic resonance imaging, PCT = procalcitonin, WBC = white blood cell.

On January 11, 2022, a critical absolute neutrophil count of 0.3 × 10^9^/L was reported, and the patient developed a high fever of 39.2°C that night. Granulocyte colony-stimulating factor (G-CSF) and leukopoietin were administered for neutropenia. Despite granulocyte-supportive treatment, high fever ranging from 39.1°C to 39.9°C persisted from January 11 to 13. Because secondary bacterial infection could not be excluded in the setting of severe neutropenia and persistent high fever, piperacillin–tazobactam was discontinued, and antimicrobial therapy was escalated to meropenem 0.5 g every 8 hours on January 13. The patient’s body temperature subsequently normalized, and the CRP level decreased from 181.7 mg/L on January 13 to 15.1 mg/L on January 19. Dynamic changes in body temperature, blood cell counts, and inflammatory markers are shown in Figures 1–5.

### 2.4. Follow-up and discharge

On January 19, after 7 days of meropenem therapy, 5 consecutive days without fever, and marked clinical and inflammatory improvement, antimicrobial therapy was de-escalated to cefixime granules 50 mg twice daily via a nasogastric tube. Fever recurred on January 21, with a maximum temperature of 38.6°C. Because recurrent infection remained a clinical concern, meropenem 0.5 g every 8 hours was reinitiated while further clinical reassessment was undertaken. The patient’s body temperature subsequently returned to normal and remained stable. Meropenem was discontinued on January 25, and the patient was discharged on January 27, 2022, in stable clinical condition.

Following discharge, the patient was followed by a clinical pharmacist via telephone at 1 month, 3 months, 6 months, and 1 year. At the most recent follow-up, the patient remained in good clinical condition, with no reported recurrence of fever.

### 2.5. Diagnostic evaluation of recurrent fever and agranulocytosis

Following the development of recurrent fever and agranulocytosis, a comprehensive diagnostic evaluation was undertaken to investigate potential infectious and noninfectious etiologies. The patient’s medication exposure, serial laboratory findings, microbiological investigations, imaging results, and evolving clinical diagnoses were systematically reviewed. Medications potentially associated with hematological adverse reactions are summarized in Table 1.

**Table 1:** Medications associated with adverse reactions.

Medication name	Dosage and administration	Medication time
Piperacillin sodium and tazobactam sodium for injection (4.5 g: 4.0 g/ 0.5 g)	4.5 g Ivgtt q8h	Dec 22, 2021–Jan 13, 2022
Meropenem for injection (0.5 g)	0.5 g Ivgtt q8h 0.5 g Ivgtt q8h	Jan 13, 2022– Jan 19, 2022 Jan 21, 2022–Jan 25, 2022
Human G-CSF injection (0.5 ml: 0.125 mg)	0.25 mg SC qd	Jan 11, 2022–Jan 15, 2022
Cefixime granules (50 mg)	50 mg NG bid	Jan 19, 2022–Jan 21, 2022

Ivgtt q8h = intravenous drip every 8 hours, G-CSF = granulocyte colony-stimulating factor, NG bid = administration via nasogastric tube twice daily, SC qd = subcutaneous injection once daily.

Pulmonary infection, megaloblastic anemia, sepsis-associated hematological abnormalities, infection-related agranulocytosis, and drug-induced agranulocytosis were all considered during the differential diagnostic process. These conditions were systematically evaluated but did not fully explain the overall clinical course.

The microbiological investigations performed during the diagnostic evaluation are summarized in Table 2. Blood cultures collected on December 21, 2021, and January 11, 2022, yielded *Staphylococcus hominis* from only a single aerobic culture bottle on each occasion, whereas the paired cultures remained negative. Moreover, the 2 isolates demonstrated different antimicrobial susceptibility profiles, suggesting contamination rather than persistent bacteremia. Urine culture demonstrated mixed bacterial growth without identification of a predominant pathogen, the intravenous catheter culture was negative, and repeat blood cultures obtained on January 13 remained sterile after 7 days of incubation. Collectively, these microbiological findings did not support sustained bacteremia or catheter-related bloodstream infection as the primary explanation for the recurrent fever.

**Table 2:** Microbiological investigations during the diagnostic evaluation of recurrent fever and agranulocytosis.

Collection date	Specimen	Culture findings	Antimicrobial susceptibility	Clinical interpretation	Impact on antimicrobial management
Dec 21, 2021	Blood cultures (2 sets)	*Staphylococcus hominis* isolated from 1 left-sided aerobic culture bottle only; all paired aerobic and anaerobic cultures remained negative after 7 days	Resistant to erythromycin only; susceptible to oxacillin and all other tested agents	Considered contamination because only a single aerobic bottle was positive and paired cultures were negative	No change in antimicrobial therapy
Jan 11, 2022	Blood cultures (2 sets)	*Staphylococcus hominis* isolated from 1 left-sided aerobic culture bottle only; all paired aerobic and anaerobic cultures remained negative after 7 days	Resistant to erythromycin, ciprofloxacin, clindamycin, moxifloxacin, and oxacillin; susceptible to vancomycin, linezolid, and teicoplanin	Considered contamination because only one bottle was positive, paired cultures were negative, and the susceptibility profile differed from the previous isolate	No change in antimicrobial therapy
Jan 12, 2022	Urine culture	Mixed growth of more than 3 bacterial species; no predominant pathogen identified	Not applicable	Mixed bacterial growth without evidence of a specific urinary pathogen	No change in antimicrobial therapy
Jan 12, 2022	Intravenous catheter culture	No bacterial growth after 3 days of incubation	Not applicable	Negative	No change in antimicrobial therapy
Jan 13, 2022	Repeat blood cultures (bilateral aerobic and anaerobic cultures)	No bacterial growth after 7 days of incubation	Not applicable	No microbiological evidence of sustained bacteremia	Supported reassessment of noninfectious causes of recurrent fever

Hematology consultations were obtained on January 12 and January 13, 2022. At the initial consultation, megaloblastic anemia with agranulocytosis was considered, and the hematology team recommended additional investigations, including repeat anemia-related tests, reticulocyte count, peripheral blood smear with neutrophil alkaline phosphatase scoring, erythropoietin measurement, and serum protein electrophoresis. Hematopoietic support with folic acid and mecobalamin in addition to G-CSF, protective isolation, and serial complete blood count monitoring were also recommended. Bone marrow aspiration was proposed if the peripheral blood cell counts continued to decline and the patient or her family consented. At the subsequent consultation, infection-related leukopenia could not be excluded, and continued antimicrobial and hematopoietic supportive treatment was recommended. Bone marrow aspiration with biopsy was to be considered if leukopenia persisted after infection control. As the neutrophil count subsequently recovered after discontinuation of piperacillin–tazobactam and administration of G-CSF and supportive treatment, bone marrow aspiration and biopsy were not subsequently pursued. Considering the temporal relationship between prolonged piperacillin–tazobactam exposure and the onset of recurrent fever and agranulocytosis, together with gradual clinical improvement after drug discontinuation and supportive treatment, a Naranjo adverse drug reaction probability scale score of 7 indicated a probable association between piperacillin–tazobactam and the observed adverse events.

## 3. Discussion

Infection is a common complication of stroke that significantly impacts prognosis, length of hospitalization, and healthcare costs.^[3–5]^ Stroke-associated pneumonia (SAP), defined as pneumonia occurring within 7 days after stroke onset,^[6]^ requires tailored antibiotic strategies based on timing of onset. According to the pneumonia in stroke consensus,^[7]^ early-onset SAP (< 72 hours) should cover common community pathogens, while late-onset SAP (72 hours to 7 days) requires broader coverage including Enterobacteriaceae and potentially *Pseudomonas aeruginosa*. Our patient developed pulmonary infection 3 days poststroke, qualifying as late-onset SAP, making piperacillin–tazobactam an appropriate empirical choice per the pneumonia in stroke consensus.

During the initial treatment period (Dec 22–30, 2021), the decreasing fever and white blood cell counts suggested therapeutic effectiveness. The concurrent rise in CRP likely reflected ongoing cerebral infarction progression, as CRP levels correlate with infarct volume.^[8]^ From December 31, 2021 onward, the patient developed recurrent fever with rising CRP but declining neutrophils, culminating in neutropenia on January 11, 2022. The persistently low procalcitonin levels (< 0.5 ng/mL) throughout the clinical course indicated mild bacterial infection severity.^[9]^ Given the broad-spectrum antimicrobial coverage of piperacillin–tazobactam, together with the available microbiological and imaging findings, other occult infections were considered less likely, although they could not be completely excluded. Therefore, the fever after December 31 was considered consistent with a probable drug-related process in the context of prolonged piperacillin–tazobactam exposure, although concurrent clinical conditions could not be completely excluded. The persistent high fever after January 11 may have been related to a possible secondary infection during severe neutropenia; however, the available clinical findings were insufficient to establish this with certainty. The defervescence following meropenem initiation on January 13 supported this interpretation. The recurrence of fever during antimicrobial de-escalation raised concern for persistent or recurrent infection but did not establish this diagnosis. The clinical response to meropenem alone was insufficient to exclude alternative infectious or noninfectious explanations.

Based on dynamic analysis of the clinical course and laboratory findings, the etiology of fever can be categorized into 3 phases:

1. Infection-associated fever (Dec 22–30, 2021): initial fever was clearly related to pulmonary infection, with an effective response to antibiotic therapy.
2. Probable drug-induced fever (Dec 31, 2021–Jan 13, 2022): persistent fever accompanied by decreasing neutrophil counts, occurring despite improved infection parameters and after excluding other infectious or noninfectious causes (e.g., malignancy, immune disorders), strongly suggests piperacillin–tazobactam-induced drug fever.
3. Secondary infection post-neutropenia (Jan 13–25, 2022): febrile episodes during neutropenia raised concern for a possible secondary infection. However, the subsequent response to meropenem alone was insufficient to definitively establish an infectious etiology.

Alternative explanations were carefully considered throughout the diagnostic process. Persistent pulmonary infection, infection-related neutropenia, sepsis-associated hematological abnormalities, and megaloblastic anemia were all included in the differential diagnosis. Serial microbiological investigations, repeated imaging studies, hematology consultations, and dynamic laboratory monitoring were performed to evaluate these possibilities. Although none of these conditions fully explained the overall temporal relationship between prolonged piperacillin–tazobactam exposure, recurrent fever, agranulocytosis, and subsequent recovery following drug withdrawal and supportive treatment, they could not be completely excluded. Therefore, the present findings should be interpreted as supporting a probable association rather than definitive causality.

The Naranjo adverse drug reaction evaluation scale^[10]^ was applied to assess causality, yielding a total score of 7, indicating a “probable” association between piperacillin–tazobactam and the observed fever and neutropenia. Detailed results are presented in Table 3.

**Table 3:** Naranjo ADR evaluation scale.

Question	Score	Rationale
Are there previous conclusive reports on this reaction?	+1	Published cases have described piperacillin–tazobactam-associated agranulocytosis and drug fever.
Did the adverse event appear after the suspected drug was administered?	+2	Fever and agranulocytosis developed after the initiation of piperacillin–tazobactam.
Did the adverse reaction improve when the drug was discontinued or a specific antagonist was administered?	+1	Fever and neutropenia improved after piperacillin–tazobactam was discontinued and supportive treatment was administered.
Did the adverse reaction reappear when the drug was readministered?	0	Rechallenge was not performed.
Are there alternative causes that could have caused the reaction?	+2	Alternative infectious and noninfectious causes were systematically evaluated but did not fully explain the overall clinical course.
Did the reaction reappear when a placebo was administered?	0	A placebo was not administered.
Was the drug detected in blood or other body fluids at a concentration known to be toxic?	**0**	Therapeutic drug monitoring was not performed.
Was the reaction more severe when the dose was increased or less severe when the dose was decreased?	0	The piperacillin–tazobactam dose was not adjusted during treatment.
Did the patient have a similar reaction to the same or a similar drug during a previous exposure?	0	No previous exposure to a similar agent or comparable reaction was documented.
Was the adverse event confirmed by objective evidence?	+1	Serial body temperature, white blood cell count, absolute neutrophil count, and inflammatory markers provided objective evidence supporting the temporal association.
Total score	7	Probable ADR

Interpretation: ≥ 9 = definite; 5–8 = probable; 1–4 = possible; ≤ 0 = doubtful.

ADR = adverse drug reaction.

Piperacillin/tazobactam (PT) is a beta-lactam/beta-lactamase inhibitor combination with a broad spectrum of antibacterial activity that includes many gram-positive organisms as well as many gram-negative bacteria. Piperacillin sodium exerts bactericidal activity by inhibiting septum formation and cell wall synthesis of susceptible bacteria. Tazobactam is a triazolylmethyl penicillanic acid sulfone which enhances and extends the antibiotic spectrum of piperacillin to beta-lactamase-producing bacteria.^[11]^ It is effective for the treatment of patients with polymicrobial infections caused by many Gram-negative, Gram-positive, and beta-lactamase-producing bacteria, such as complicated nosocomial and intra-abdominal infections, and also is regarded as empiric therapy for patients who present with fever and neutropenia.^[12]^

According to the prescribing information for piperacillin–tazobactam, fever is a common adverse reaction (≥ 1/100–< 1/10), while leukopenia is uncommon (≥ 1/1000–< 1/100) and agranulocytosis is rare (≥ 1/10000–< 1/1000).^[13]^ However, literature reports indicate hematological adverse events are not infrequent. Reports have described bicytopenia at lower cumulative doses,^[14]^ including cases in special populations such as pregnant^[15]^ and postpartum patients.^[16]^ Additionally, a further case report documented concurrent hemolytic anemia and thrombocytopenia induced by PT.^[17]^ A systematic review of 62 cases identified hemolytic anemia (40.3%), thrombocytopenia (37.1%), and neutropenia (19.4%) as predominant hematological toxicities.^[1]^ Drug-induced fever may occur through various mechanisms, including hypersensitivity reactions.^[18]^ Most reported cases of PT-associated fever coincide with hematological abnormalities.^[12,19]^ Recent studies have further confirmed this association, with 1 case demonstrating fever and abnormal laboratory parameters resolving after drug discontinuation.^[20]^

Evidence suggests strong correlations between adverse effects and treatment duration/cumulative dose. Studies indicate neutropenia incidence increases with cumulative piperacillin exposure.^[2]^ Risk factors include longer duration, combination therapy, and specific laboratory parameters.^[21]^ Neutropenia is considered to be associated with bone marrow suppression, typically occurring approximately 2 weeks following the initiation of PT therapy.^[1]^ Comparative analyses demonstrate higher adverse event rates with prolonged PT therapy versus other antibiotics.^[22]^

## 4. Limitations

This case report has several limitations. First, rechallenge with piperacillin–tazobactam was not performed because of ethical and patient-safety considerations; therefore, a definitive causal relationship cannot be established. Second, although alternative infectious and noninfectious etiologies were systematically evaluated, they could not be completely excluded. Third, the patient’s clinical improvement occurred after multiple concurrent interventions, including discontinuation of piperacillin–tazobactam, modification of antimicrobial therapy, administration of G-CSF, and supportive treatment, making it difficult to determine the relative contribution of each intervention. Finally, as this is a single-case report, the findings should be interpreted cautiously and require confirmation in larger observational studies.

In conclusion, prolonged piperacillin–tazobactam therapy warrants vigilance for delayed drug fever and hematological toxicity. When persistent or recurrent fever is accompanied by a declining neutrophil count during treatment, piperacillin–tazobactam should be considered a potential cause after alternative infectious and noninfectious etiologies have been carefully evaluated and reasonably excluded. In the present case, the temporal relationship and a Naranjo score of 7 supported a probable, rather than definitive, association between piperacillin–tazobactam and the observed adverse events. Early recognition, timely discontinuation of the suspected drug when clinically appropriate, and close hematological monitoring may help prevent further complications and unnecessary diagnostic or therapeutic interventions.

## Author contributions

**Investigation:** Wei Jin.

**Visualization:** Yuhua Wu.

**Writing – original draft:** Lijiang Wang.

**Writing – review & editing:** Menglu Zhu.
